# Cell biology of protein–lipid conjugation

**DOI:** 10.1247/csf.23016

**Published:** 2023-04-06

**Authors:** Jun-ichi Sakamaki, Noboru Mizushima

**Affiliations:** 1 Department of Biochemistry and Molecular Biology, Graduate School of Medicine, The University of Tokyo, 7-3-1 Hongo, Bunkyo-ku, Tokyo 113-0033, Japan

**Keywords:** lipid, lipidation, membrane, organelle, protein modification

## Abstract

Protein–lipid conjugation is a widespread modification involved in many biological processes. Various lipids, including fatty acids, isoprenoids, sterols, glycosylphosphatidylinositol, sphingolipids, and phospholipids, are covalently linked with proteins. These modifications direct proteins to intracellular membranes through the hydrophobic nature of lipids. Some of these membrane-binding processes are reversible through delipidation or by reducing the affinity to membranes. Many signaling molecules undergo lipid modification, and their membrane binding is important for proper signal transduction. The conjugation of proteins to lipids also influences the dynamics and function of organellar membranes. Dysregulation of lipidation has been associated with diseases such as neurodegenerative diseases. In this review, we first provide an overview of diverse forms of protein–lipid conjugation and then summarize the catalytic mechanisms, regulation, and roles of these modifications.

## Introduction

**Fig. 1:**
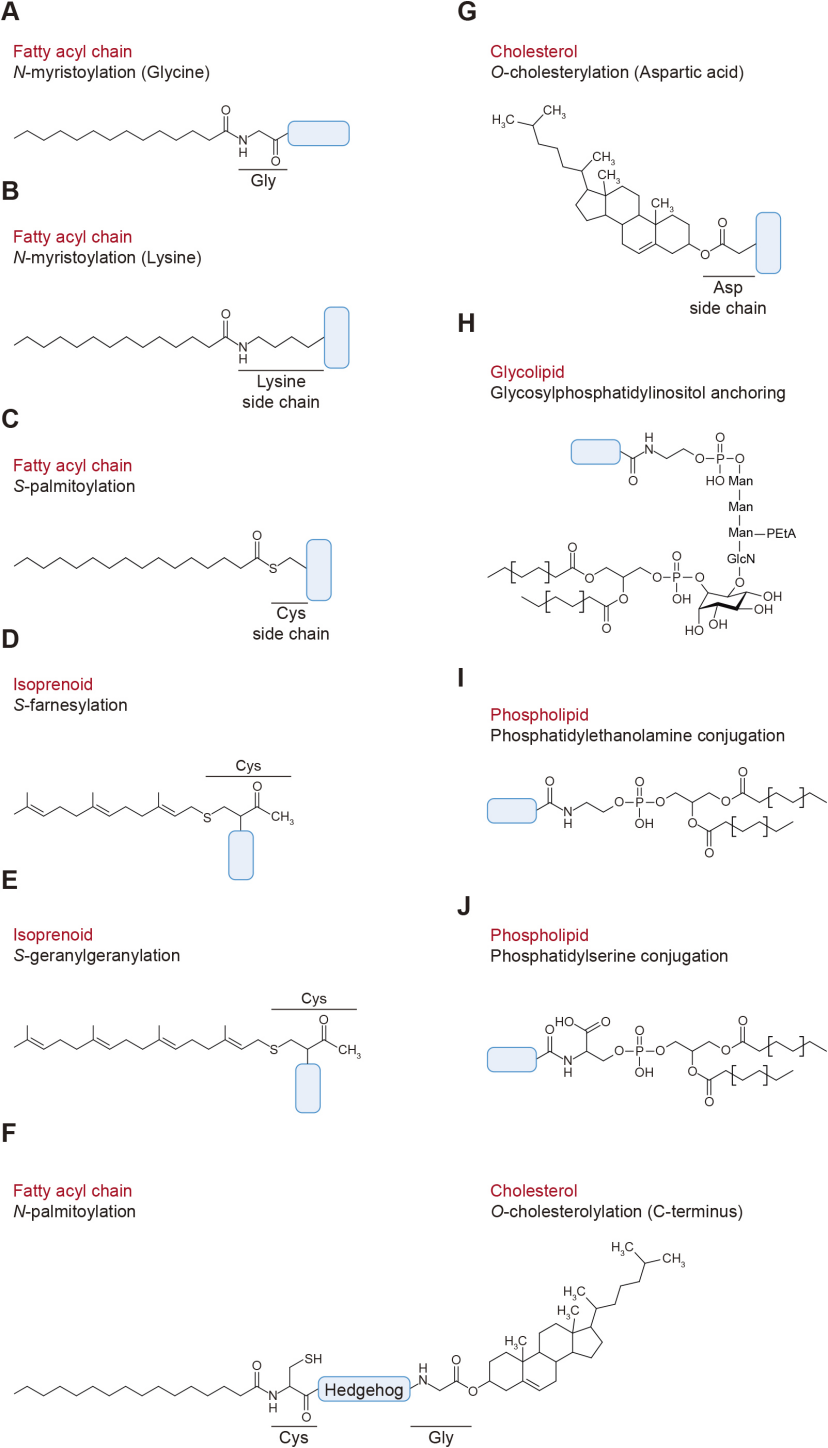
Diverse types of protein–lipid conjugation A. Glycine *N*-myristoylation. Myristic acid forms an amide bond with glycine. B. Lysine *N*-myristoylation. Myristic acid is covalently linked to the side chain of lysine. C. Cysteine *S*-palmitoylation. Palmitic acid forms a thioester linkage with the side chain of cysteine. D and E. Cysteine *S*-prenylation. The farnesyl (D) or geranylgeranyl (E) group is covalently bound to the side chain of cysteine. F. Hedgehog is modified with palmitic acid and cholesterol at its N- and C- termini, respectively. G. The hydroxy group of cholesterol is covalently linked to the side chain of aspartic acid within Smoothened (SMO). H. The phosphoethanolamine moiety of glycosylphosphatidylinositol is covalently linked to the carboxyl group of the C-terminal amino acid via an amide bond. I and J. The carboxyl group of the C-terminal glycine residue of ubiquitin family proteins is covalently attached to the amino group of phosphatidylethanolamine (I) and phosphatidylserine (J).

Numerous proteins are associated with cellular membranes. Besides having transmembrane domains or binding to other membrane proteins, direct conjugation to membrane lipids is one of the common mechanisms through which proteins associate with cellular membranes. Covalent conjugation between proteins and lipids is widely observed in eukaryotes and is involved in many biological processes. Various types of lipids, including fatty acids, isoprenoids, sterols, glycosylphosphatidylinositol, sphingolipids, and phospholipids, can covalently conjugate to proteins (Fig. 1) (Chen *et al.*, 2018; Resh, 2013). Some proteins receive multiple lipid modifications (e.g., H- and N-Ras, YKT6, and Hedgehog, see below). These lipids are linked to the N- or C-terminus or a side chain of an internal amino acid in proteins. Attachment of lipids generally increases the hydrophobicity of proteins and directs them to specific cellular membrane compartments or domains. Well-known lipid modifications include myristoylation, palmitoylation, farnesylation, geranylgeranylation, and glycosylphosphatidylinositol (GPI) anchoring. Although these modifications are already textbook knowledge, novel substrates and physiological functions have continued to be discovered in recent years. In addition, cholesterol has been added to this list of lipids that can be used for protein modification. Furthermore, recent studies demonstrated that ubiquitin and ubiquitin-like proteins can be conjugated to phospholipids. In this review, we summarize the different types of protein–lipid conjugation, the catalyzing and regulatory mechanisms of these modifications, and their biological roles.

## Myristoylation

**Fig. 2:**
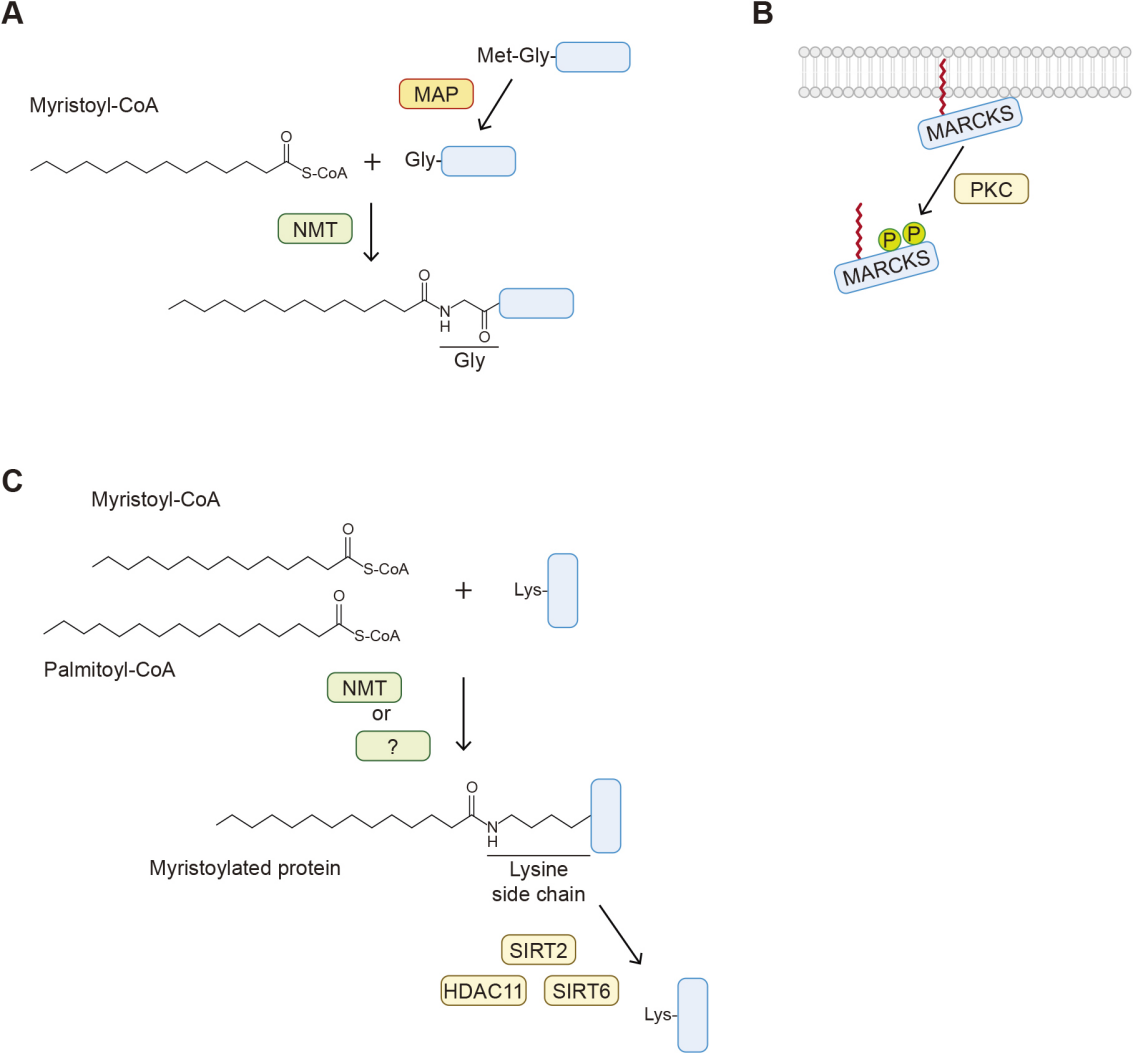
Overview of *N*-myristoylation A. The mechanism of glycine *N*-myristoylation. The initiating methionine is cleaved by methionine amino peptidase (MAP), and myristic acid is covalently attached to the exposed glycine residue by *N*-myristoyl transferase (NMT) proteins. Myristoyl-CoA is the myristic acid donor. B. Myristoylated alanine-rich protein kinase C substrate (MARCKS) is associated with membranes through *N*-myristoylation and its positively charged basic amino acids. Phosphorylation within the basic amino acids by protein kinase C (PKC) causes the dissociation of MARCKS from membranes. C. The mechanism of lysine *N*-fatty acylation. Fatty acyl groups such as myristic and palmitic acids are covalently linked to the side chain of lysine residues by NMT (lysine *N*-myristoylation) and unknown enzymes. SIRT2, SIRT6, and HDAC11 remove fatty acyl groups from proteins.

Covalent attachment of myristic acid, a 14-carbon saturated fatty acid, occurs mainly at the glycine residue at position 2 of a protein carrying the consensus sequence Met-Gly-X-X-X-Ser/Thr (X: any amino acid) (Fig. 1A) (Farazi *et al.*, 2001; Udenwobele *et al.*, 2017; Wang *et al.*, 2021; Yuan *et al.*, 2020). Generally, *N*-myristoylation occurs co-translationally. First, the initiating methionine is removed by methionine amino peptidase (Fig. 2A). Then, *N*-myristoyl transferases (NMTs; NMT1 and NMT2 in human) transfer myristate from the acyl donor myristoyl-CoA to the amino group of the exposed glycine residue. One of the major roles of *N*-myristoylation is to assist with membrane localization. The hydrophobicity of myristate is generally insufficient to anchor proteins to membranes; it requires an additional membrane association property, such as a cluster of positively charged basic amino acids that interact with negatively charged phospholipids in membranes (e.g., in myristoylated alanine-rich protein kinase C substrate [MARCKS]) (Kim *et al.*, 1994), hydrophobic residues (e.g., in ADP ribosylation factor 1 (ARF1)) (Antonny *et al.*, 1997), and palmitoylation (e.g., in Src family tyrosine kinases) (Shenoy-Scaria *et al.*, 1994). One exception is the *N*-myristoylation of protein kinase A (PKA) catalytic subunits, which is sufficient for localization to the plasma membrane (Xiong *et al.*, 2021). The membrane association of *N*-myristoylated proteins is reversed by diminishing these membrane association properties. For example, phosphorylation within the basic amino acid cluster by protein kinase C (PKC) causes the dissociation of MARCKS from membranes (Fig. 2B) (Thelen *et al.*, 1991).

Glycine *N*-myristoylation is irreversible under physiological conditions. However, invasion plasmid antigen J (IpaJ), a *Shigella flexneri* effector protein, possesses cysteine protease activity that cleaves between the *N*-myristoylated glycine and the amino acid at position 3 (Burnaevskiy *et al.*, 2013; Burnaevskiy *et al.*, 2015). Although IpaJ can cleave various *N*-myristoylated proteins *in vitro*, it specifically cleaves the Golgi-localized ARF and ARF-like (ARL) family of GTPases *in vivo* during *Shigella* infection.

When proteins fail to undergo *N*-myristoylation, they are degraded by a glycine-specific N-degron pathway (Timms *et al.*, 2019). This quality control mechanism involves the ubiquitin ligase complexes Cul2^ZYG11B^ and Cul2^ZER2^, which ubiquitinate proteins that expose the second glycine residue but fail to undergo N-myristoylation for degradation by the proteasome.

Post-translational *N*-myristoylation is also observed during apoptosis (Vilas *et al.*, 2006; Zha *et al.*, 2000). For example, in the cleavage of BH3 interacting domain death agonist (BID)—a proapoptotic BH3-only protein—by caspase-8, the consensus motif in the C-terminal p15 fragment (tBID) is exposed and myristoylated (Zha *et al.*, 2000). This triggers the translocation of BID to mitochondria and ultimately cell death.

## Lysine *N*-fatty acylation

Various fatty acyl groups, including myristoyl, palmitoyl, and butyryl groups, are also covalently attached to the ε-amino group of the side chain of lysine residues (Fig. 1B). The substrates include ARF6 (Kosciuk *et al.*, 2020), R-Ras2 (Zhang *et al.*, 2017), K-Ras4a (Jing *et al.*, 2017), A-kinase anchoring protein 12 (AKAP12) (Bagchi *et al.*, 2022), tumor necrosis factor α (TNFα) (Jiang *et al.*, 2013), and serine hydroxymethyltransferase 2 (SHMT2) (Cao *et al.*, 2019). These modifications regulate membrane localization (Jing *et al.*, 2017; Kosciuk *et al.*, 2020; Zhang *et al.*, 2017) and protein secretion (Jiang *et al.*, 2013).

Lysine *N*-myristoylation is also catalyzed by NMT1 and NMT2 (Dian *et al.*, 2020; Kosciuk *et al.*, 2020), whereas the enzymes catalyzing lysine palmitoylation and butyrylation are unknown (Fig. 2C). Unlike glycine *N*-myristoylation, lysine *N*-myristoylation is reversible; fatty acyl deconjugation is catalyzed by lysine deacetylase family proteins, including sirtuin 2 (SIRT2) (Jing *et al.*, 2017), SIRT6 (Jiang *et al.*, 2013; Zhang *et al.*, 2017), and histone deacetylase 11 (HDAC11) (Fig. 2C) (Bagchi *et al.*, 2022; Cao *et al.*, 2019). SIRT6 possesses a large hydrophobic pocket within which long-chain fatty acyl groups fit and preferentially remove these acyl groups rather than the acetyl group. Fatty acyl deconjugation liberates signaling molecules and thus regulates signal transduction. Collectively, reversible lysine *N*-fatty acylation has further expanded the known physiological significance of fatty acylation of proteins.

## Palmitoylation

**Fig. 3:**
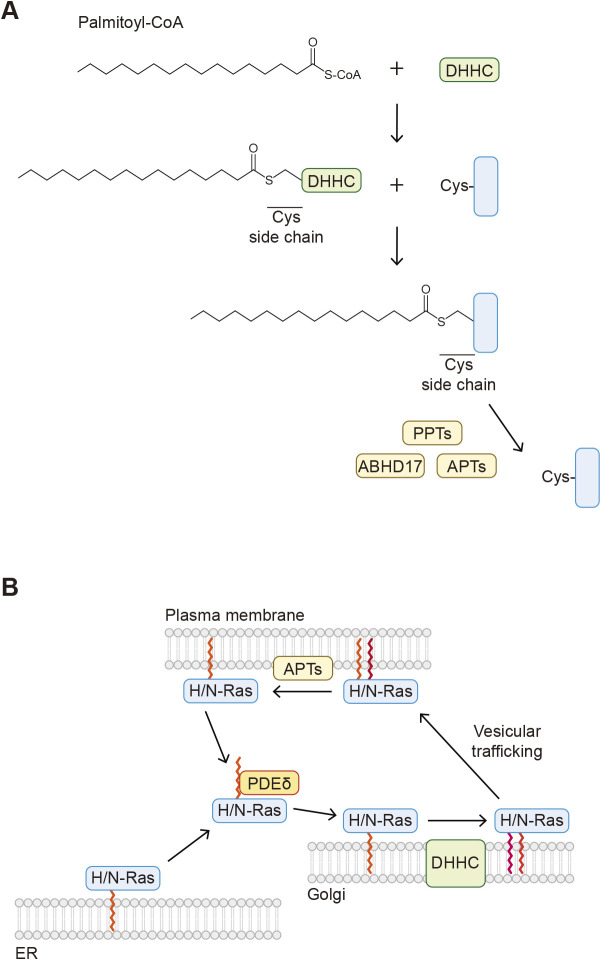
Overview of *S*-palmitoylation A. The mechanism of cysteine *S*-palmitoylation. The palmitoyl group of palmitoyl-CoA forms an intermediate with the catalytic cysteine residue of the Asp-His-His-Cys (DHHC) protein and then is transferred to a cysteine residue in a substrate protein. B. Farnesylated H- and N-Ras proteins localized to the ER are dissociated from the membranes by PDEδ and transported to the Golgi. H-/N-Ras proteins are then modified with *S*-palmitoylation by DHHC and localized to the plasma membrane through vesicle trafficking. H-/N-Ras proteins are depalmitoylated by acyl-protein thioesterase (APT) and detached from the membranes.

There are three types of protein palmitoylation: *S*-, *O*-, and *N*-palmitoylation (Chen *et al.*, 2018; Jin *et al.*, 2021). The most common type is *S*-palmitoylation, in which the 16-carbon saturated fatty acid palmitic acid is conjugated to a cysteine residue of a protein (Fig. 1C). In the presence of palmitoyl-CoA as an acyl donor, *S*-palmitoylation is catalyzed by Asp-His-His-Cys (DHHC) family proteins. There are 23 DHHC family proteins in humans, all of which possess the conserved catalytic DHHC sequence and transmembrane domains. These enzymes are localized to the Golgi, the endoplasmic reticulum (ER), or the plasma membrane. The process of *S*-palmitoylation includes two steps. The palmitoyl group of palmitoyl-CoA is first conjugated to the catalytic cysteine residue of the DHHC proteins (autopalmitoylation) and is then transferred to a cysteine residue of a substrate protein (Fig. 3A).

One of the major roles of *S*-palmitoylation is to control the membrane localization of proteins. Proteins that undergo *S*-palmitoylation may also be subjected to other lipid modifications such as *N*-myristoylation (e.g., in Src family tyrosine kinases), *S*-prenylation (e.g., Ras, YKT6, see below), and cholesterylation (e.g., Hedgehog, see below). An important characteristic of *S*-palmitoylation is its reversibility, which can change hydrophobicity and membrane localization. Depalmitoylation is catalyzed by different types of enzymes, including palmitoyl protein thioesterases (PPT1 and PPT2) and acyl-protein thioesterases (APT1 and APT2). PPT1 and PPT2 are localized mainly to lysosomes and are involved in depalmitoylation of transmembrane proteins. The importance of these enzymes is highlighted by patients with mutations in the *PPT1* gene (also known as ceroid lipofuscinosis neuronal 1 [*CLN1*]) (Vesa *et al.*, 1995); these patients exhibit a fatal inherited neurodegenerative lysosomal storage disease. Similarly, mice lacking *Ppt1* or *Ppt2* exhibit neuronal ceroid lipofuscinosis (Gupta *et al.*, 2001). Cytosolic APT1 and APT2 themselves are *S*-palmitoylated and localized to membranes. These enzymes are involved in depalmitoylation of cytosolic proteins, including H-Ras and the α subunit of G proteins (Duncan and Gilman, 1998). In addition to these four enzymes, alpha/beta hydrolase domain-containing protein 17 (ABHD17), a protein localized to the plasma membrane by S-palmitoylation, is also involved in depalmitoylation of several proteins, including N-Ras and PSD95 (Lin and Conibear, 2015).

Reversible *S*-palmitoylation allows proteins to shuttle between different membrane compartments in response to intrinsic and extrinsic stimuli (Chen *et al.*, 2018). For example, H- and N-Ras are modified with palmitoylation and farnesylation (see below) and anchored to membranes. Upon depalmitoylation, H- and N-Ras dissociate from the membranes. H- and N-Ras undergo continuous palmitoylation and depalmitoylation cycles and shuttle between the plasma membrane and the Golgi (Rocks *et al.*, 2005), which requires proper Ras signaling (Fig. 3B).

Some of the soluble *N*-ethylmaleimide-sensitive-factor attachment protein receptor (SNARE) proteins, which mediate the fusion of two membranes, undergo *S*-palmitoylation (Prescott *et al.*, 2009; Kriegenburg *et al.*, 2019). Synaptobrevin homolog YKT6 is modified by both palmitoylation and farnesylation (Dietrich *et al.*, 2005; Fukasawa *et al.*, 2004; McNew *et al.*, 1997). YKT6 is an autophagosomal SNARE protein and mediates autophagosome–lysosome (or autophagosome–vacuole) fusion (Bas *et al.*, 2018; Matsui *et al.*, 2018). The lipidation status of YKT6 regulates its localization and function. Doubly lipidated YKT6 is stably anchored to membranes, whereas the depalmitoylated form is localized to the cytosol. Upon depalmitoylation, the farnesyl moiety inserts into the hydrophobic groove formed between the longin domain and its SNARE core of YKT6, which locks it in a soluble and closed inactive conformation (Kriegenburg *et al.*, 2019; Wen *et al.*, 2010).

Transmembrane proteins are also subjected to *S*-palmitoylation. This regulates subcellular trafficking and targeting of transmembrane proteins. In addition, *S*-palmitoylation of membrane proteins directs them to lipid rafts, a microdomain in the plasma membrane enriched with saturated lipids, cholesterol, and sphingolipids (Charollais and Van Der Goot, 2009).

Because lipidated proteins are directed to membranes, it is conceivable that lipidation can alter the properties of membranes themselves. The protein depalmitoylases APT2 undergoes *S*-palmitoylation and binds to membranes. Molecular dynamics simulation predicts that membrane-bound APT2 deforms membranes to facilitate the removal of acyl chains conjugated to other proteins (Abrami *et al.*, 2021).

The process of *O*-palmitoylation, in which palmitate covalently binds to the hydroxy group of serine or threonine, is observed in histone H4 protein. Lysophosphatidylcholine acyltransferase 1 (LPCAT1) transfers palmitate to histone H4, and this modification regulates global mRNA synthesis (Zou *et al.*, 2011).

Palmitate is also conjugated to the N-terminal amino group (*N*-palmitoylation) of the N-terminus of hedgehog protein (Hh) (Pepinsky *et al.*, 1998). After cleavage of the signal peptide, palmitate is attached to Hh by the action of hedgehog acyltransferase (HHAT) (Fig. 1F; see also Fig. 5) (Buglino and Resh, 2008). HHAT is a member of the membrane-bound *O*-acyltransferase (MBOAT) family that localize to the ER and palmitoylation occurs inside the ER (Matevossian and Resh, 2015). The C-terminus of Hh is modified with cholesterol (see below).

Acylation is not limited to myristoylation and palmitoylation. Other fatty acids, including palmitoleic acid (*O*-palmitoleoylation) and caprylic (octanoic) acid (*O*-octanoylation), also covalently bind to proteins. Wnt is *O*-palmitoleoylated by the acyltransferase Porcupine, and this modification regulates its trafficking and secretion (Takada *et al.*, 2006). Ghrelin, an appetite-stimulating peptide hormone produced by the stomach, is modified with octanoate; *O*-octanoylation of ghrelin is catalyzed by ghrelin O-acyltransferase (GOAT) and is required for its function (Yang *et al.*, 2008). Porcupine and GOAT are also members of the MBOAT family and modify Wnt and Ghrelin in the ER like HHAT.

## Prenylation (farnesylation and geranylgeranylation)

**Fig. 4:**
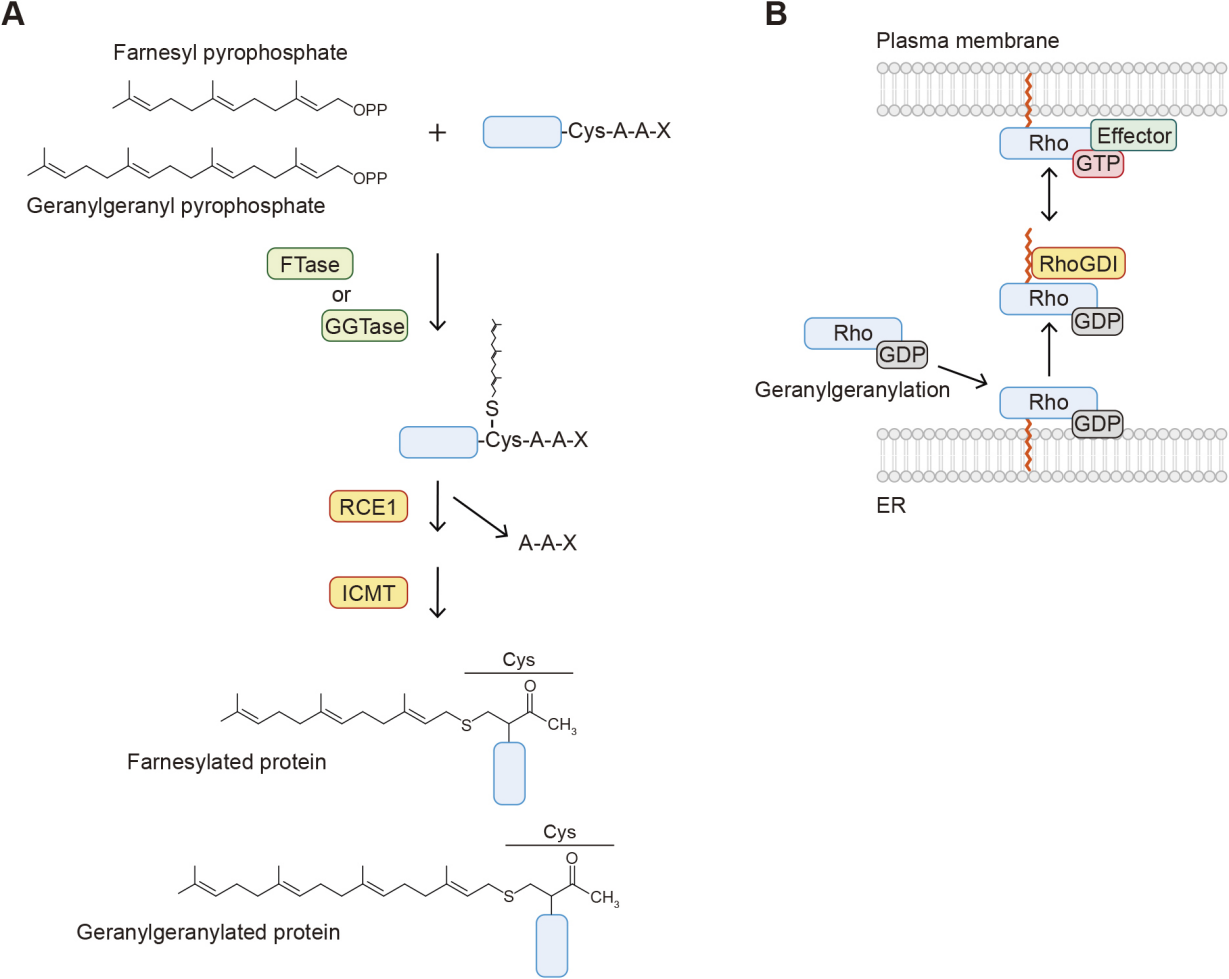
Overview of *S*-prenylation A. The mechanism of *S*-prenylation. The farnesyl and geranylgeranyl groups of farnesyl pyrophosphate and geranylgeranyl pyrophosphate are covalently linked to the side chain of the cysteine residue within the CAAX motif. The AAX peptides are then removed by Ras-converting CAAX endopeptidase 1 (RCE1), and the C-terminal cysteine residue is methylated by isoprenylcysteine carboxylmethyltransferase (ICMT). B. Geranylgeranylated Rho proteins are dissociated from membranes by the RhoGDI protein. The GTP-bound Rho activates downstream effector proteins localized to the plasma membrane. RhoGDI sequesters Rho in the cytosol and locks it in the GDP-bound state.

Attachment of 15-carbon farnesyl and 20-carbon geranylgeranyl isoprenoids to the side chain of cysteine is also a common lipid modification of proteins (Fig. 1D and E) (Wang and Casey, 2016). It occurs on proteins carrying the consensus CAAX motif (where A is an aliphatic residue). The isoprenoids form a thioester linkage with the side chain of the cysteine residue within the motif. This modification is catalyzed by farnesyltransferase (FTase) and geranylgeranyltransferases (GGTase 1, 2, and 3) in the presence of farnesyl pyrophosphate and geranylgeranyl pyrophosphate, which are synthesized through the mevalonate pathway (Fig. 4A). FTase and GGTases consist of one α and one β subunit. FTase and GGTase 1 share the same α subunit (FNTA/PTAR2) but include different β subunits (FNTB and PGGT1B). GGTase 2 consists of the α subunit RabGGTA (PTAR3) and the β subunit RabGGTB. Recently, GGTase 3 was identified and found to possess the PTAR1 α subunit and RabGGTB (Kuchay *et al.*, 2019). Position X within the motif determines which isoprenoid is attached. When X is serine, methionine, glutamine or cysteine, proteins are targeted by FTase, whereas GGTase 1 recognizes CAAX proteins with X being leucine or isoleucine (Casey *et al.*, 1991; Moores *et al.*, 1991; Yokoyama *et al.*, 1991). The reactivity of FTase and GGTase 1 is determined by the hydrophobicity and structure of position X (Hartman *et al.*, 2005). However, these enzymes have some flexibility in substrate specificity, and certain proteins (e.g., RhoB) are modified with both types of isoprenoids. Once an isoprenoid is attached, the modified proteins are subsequently processed by two enzymes localized to the ER membranes. The AAX residues are cleaved by Ras converting CAAX endopeptidase 1 (RCE1), followed by methylation at the C-terminal residue by isoprenylcysteine carboxylmethyltransferase (ICMT) (Fig. 4A).

The role of prenylation is also the regulation of protein localization and trafficking. Because the processing of prenylated proteins occurs on the ER and prenylation is irreversible, their trafficking between membrane compartments is mediated by isoprene-binding proteins or chaperones. As mentioned above, Ras and YKT6 are modified with both *S*-palmitoylation and *S*-farnesylation. During the palmitoylation/depalmitoylation cycles, farnesylated Ras is captured by phosphodiesterase-δ (PDEδ) (Fig. 3B). PDEδ functions as a solubilizing factor of farnesylated Ras and enhances effective diffusion of depalmitoylated Ras in the cytosol (Chandra *et al.*, 2011). The Rho family of small GTPases, including Rho, Rac, and Cdc42, are geranylgeranylated and anchored to the plasma membrane, where the GTP-bound active form exerts its function (e.g., cytoskeletal reorganization) by recruiting effector proteins. The geranylgeranylated Rho proteins are extracted from the membranes by RhoGDI. RhoGDI binds to Rho proteins, and the hydrophobic groove of RhoGDI accommodates the geranylgeranyl group within Rho proteins, which sequesters Rho proteins in the cytosol and locks them in the GDP-bound state (Fig. 4B) (Hoffman *et al.*, 2000).

## Cholesterylation

To date, two proteins, hedgehog (Hh) and smoothened (SMO), are known to covalently bind to cholesterol. Both Hh and SMO are components of Hh signaling, a pathway important for animal development (Briscoe and Therond, 2013; Kong *et al.*, 2019).

**Fig. 5:**
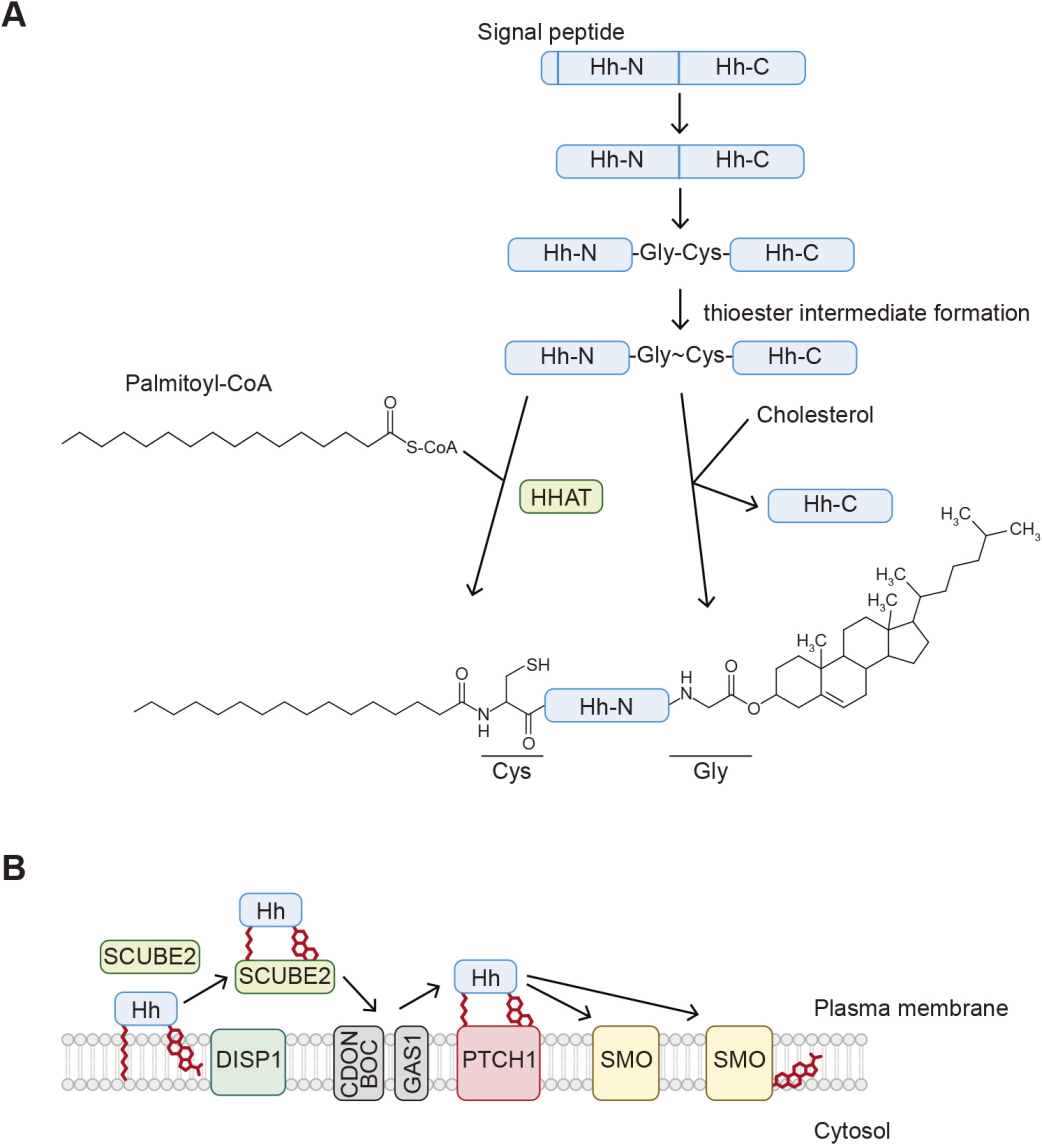
Overview of *O*-cholesterylation A. Both *N*-palmitoylation and *O*-cholesterylation of hedgehog proteins. The N-terminal signal peptide is cleaved by a signal peptidase. The exposed cysteine residue is modified through *N*-palmitoylation by hedgehog acyltransferase (HHAT), while *O*-cholesterylation of hedgehog (Hh) is coupled with its autocleavage between glycine and cysteine residues. The C3 hydroxy group of cholesterol forms an ester bond with the carboxy group of the glycine residue. (The thioester bond formed is shown as “~” here.) B. Hh that is modified through *N*-palmitoylation and *O*-cholesterylation localizes to the outside surface of the plasma membrane. Hh is released from the membrane by the action of Dispatched 1 (DISP1) and signal peptide-CUB-epidermal growth factor-like domain-containing protein 2 (SCUBE2). The transfer of Hh to its receptor Patched 1 (PTCH1) is mediated by the coreceptors cell adhesion molecule-related/down-regulated by oncogenes (CDON)/brother of CDO (BOC) and growth arrest specific 1 (GAS1). This releases the inhibition of SMO by PTCH1 and activates the downstream signaling. Hh also promotes cholesterylation of Smoothened (SMO). This modification is important for its localization to cilia and function.

Hedgehog proteins (Hh; sonic hedgehog [Shh], Indian hedgehog [Ihh], and desert hedgehog [Dhh] in mammals) are morphogens covalently modified with palmitate and cholesterol at their N- and C-termini in the secretory pathway, respectively (Figs. 1F and 5A). Cholesterol conjugation of Hh is coupled with autocleavage mediated by its C-terminal domain (Fig. 5A) (Porter *et al.*, 1996). After the signal peptide is removed in the ER, this autocleavage occurs between the glycine and cysteine residues, forming a thioester intermediate between the sulfhydryl group of the cysteine side chain and the carboxy group of glycine. The C3 hydroxy group of cholesterol acts as the nucleophile that attacks this thioester intermediate and forms an ester bond with the carboxy group of the glycine residue, releasing the C-terminal fragment of Hh. As mentioned earlier, the N-terminal Hh fragment is modified with palmitoylation. The resulting dual-lipidated Hh is associated with the membrane because of its hydrophobicity. Two solubilizing factors, Dispatched 1 (DISP1) and signal peptide-CUB-epidermal growth factor-like domain-containing protein 2 (SCUBE2), recognize lipidated Hh and mediate the secretion of Hh (Fig. 5B) (Creanga *et al.*, 2012; Petrov *et al.*, 2020; Tukachinsky *et al.*, 2012; Wierbowski *et al.*, 2020). This process depends on the sodium antiporter activity of DISP1. The coreceptors cell adhesion molecule-related/down-regulated by oncogenes (CDON)/brother of CDO (BOC) and growth arrest specific 1 (GAS1) then transfer lipidated Hh to its receptor Patched 1 (PTCH1). The palmitoylated N-terminus of Hh is inserted into a cavity of the PTCH1 extracellular domains (Qi *et al.*, 2018), which releases the inhibition of SMO by PTCH1 and activates the downstream signaling.

SMO is a GPCR-related protein that is repressed by PTCH1 and transduces the Hh signaling upon Hh binding to PTCH1 (Fig. 5B). A click chemistry-based proteomic analysis using a cholesterol analog revealed that SMO itself is also covalently modified with cholesterol (Xiao *et al.*, 2017). In contrast to Hh, cholesterol is attached to an aspartic acid of SMO via an ester bond (Fig. 1G). This covalent conjugation forms between the C3 hydroxy group of cholesterol and the carboxy group of the side chain of the aspartic acid. The reaction mechanism of SMO cholesterylation is still unknown. Cholesterylation of SMO is suppressed by PTCH1 and promoted by Hh. This modification is important for its proper localization to cilia and its function, as the cholesterylation site mutant of SMO fails to localize to cilia and activate the downstream signaling.

## GPI anchoring

Glycosylphosphatidylinositol (GPI) is a complex glycolipid that is covalently linked to the C-terminus of proteins (Kinoshita, 2020). GPI-anchored proteins (GPI-APs) are directed to the outside surface of the plasma membrane. GPI consists of phosphatidylinositol (PI), glucosamine, three mannose residues, and phosphoethanolamine. It can be further modified with monosaccharides, including *N*-acetylgalactosamine, galactose, sialic acid, and mannose. The amino group of phosphoethanolamine forms an amide bond with the carboxy group of the ω amino acid (the GPI attachment site) (Fig. 1H). GPI-APs possess a 20–30-amino acid signal peptide that extends from the ω + 1 position. This signal peptide contains approximately 10 hydrophilic and 20 hydrophobic residues, and the ω position is typically an amino acid with a small side chain.

GPI precursors are assembled by eleven sequential reactions in the cytosolic and luminal sides of the ER (Kinoshita, 2020). During these reactions, glucosamine, mannose, acyl groups, and phosphoethanolamine moieties are attached to PI. The N-terminal ER targeting signal sequence of a substrate protein is first cleaved upon its translocation to the ER. The GPI transamidase complex then cleaves the substrate protein between the ω and ω + 1 amino acids and attaches the substrate to GPI in the ER. GPI moiety is remodeled in the ER lumen. GPI-APs are then transported to the Golgi via COPII vesicles and further subjected to GPI remodeling. After remodeling in the Golgi, it is transported to the plasma membrane.

GPI-APs include adhesion molecules, receptors, and enzymes and can be localized to specific domains of the plasma membrane such as membrane rafts. In polarized cells, GPI-modified proteins can be localized exclusively to the apical or basolateral side. GPI-APs also form nanoclusters upon the activation of the integrin pathway, which functions in membrane receptor signaling. The actomyosin machinery and vinculin mediate GPI-AP nanoclustering, and this nanoclustering is required for integrin functions such as cell spreading and migration (Kalappurakkal *et al.*, 2019).

GPI moiety can be cleaved by phospholipases such as GDE2 (Park *et al.*, 2013), GDE3 (van Veen *et al.*, 2017), and PGAP6 (Lee *et al.*, 2016), thus releasing the GPI-APs from membranes. These GPI-specific phospholipases target specific GPI-APs. For example, RECK (reversion-inducing cysteine-rich protein with Kazal motifs), a GPI-anchored protease inhibitor, and glypican 6 are released from membranes by GDE2 (Matas-Rico *et al.*, 2016; Park *et al.*, 2013). The release of these proteins is important for neuronal differentiation and promotes neuroblastoma differentiation, respectively.

## Protein-bound ceramides

Specific types of ceramides, a class of sphingolipid, are covalently attached to surface proteins of corneocytes, terminally differentiated keratinocytes (Marekov and Steinert, 1998). These surface proteins, termed cornified envelope proteins, form an ester bond with the ω-hydroxy group of ω-hydroxyceramides which consist of a sphingosine and a ω-hydroxy fatty acid (Kihara, 2016). Transglutaminase 1 catalyzes the attachment of ω-hydroxyceramide to glutamine residues of cornified envelope proteins including involucrin (Nemes *et al.*, 1999). Protein-bound ceramides form nonenzymatically (Takeichi *et al.*, 2020). Lipoxygenases and short chain dehydrogenase/reductase family 9C member 7 (SDR9C7) generate a chemically reactive derivative of ω-O-acylceramide which is composed of a sphingosine and an esterified ω-hydroxy fatty acid. This ω-O-acylceramide derivative nonenzymatically couples with proteins. Covalent binding of epidermal ceramides to cornified envelope proteins functions to connect lipid lamellae and corneocytes and is important for skin barrier formation.

## Phospholipid conjugation

**Fig. 6:**
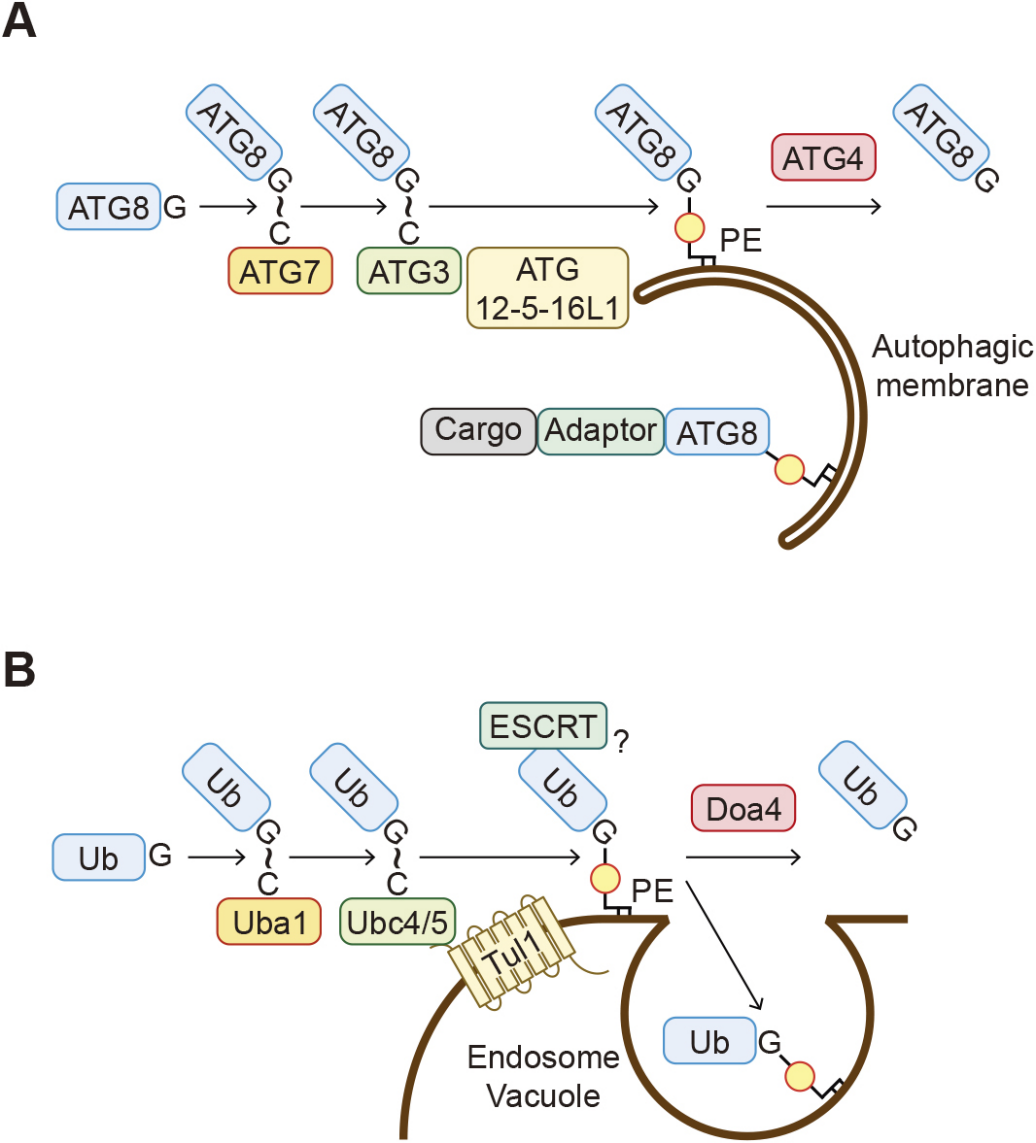
Conjugation of the ubiquitin family proteins to phospholipids A. ATG8 proteins are covalently linked to phosphatidylethanolamine (PE) in the autophagic membranes through the sequential reactions involving ATG7, ATG3, and the ATG12–ATG5–ATG16L1 complex. Cargo proteins are incorporated into the autophagic membranes, often via adaptor proteins. ATG8-PE is cleaved by ATG4 proteins and released from membranes. (The thioester bond formed is shown as “~” here.) B. Ubiquitin is covalently linked to PE in the endosomal and vacuolar membranes by the ubiquitin system enzymes, including Uba1, Ubc4/5, and Tul1. Ub-PE is turned over by either deubiquitination by Doa4 or degradation in the vacuole after being incorporated into intraluminal vesicles. Ub-PE recruits the ESCRT components to membranes *in vitro* and may be involved in intraluminal vesicle formation. (The thioester bond formed is shown as “~” here.)

Phospholipid–protein conjugation was first characterized in ATG8 family proteins (Atg8 in yeast and LC3s and GABARAPs in mammals), which are ubiquitin-like proteins involved in autophagy. Autophagy is a process in which a double-membrane structure called the autophagosome engulfs part of the cytoplasm for degradation in lysosomes or the vacuole. During this process, ATG8 is conjugated to the phospholipid phosphatidylethanolamine (PE) in autophagosomes by the action of two ubiquitin-like systems (Figs. 1I and 6A) (Ichimura *et al.*, 2000; Kabeya *et al.*, 2004; Mizushima *et al.*, 1998; Nakatogawa, 2013). ATG8 is first processed at the C-terminus by the cysteine protease ATG4 (Atg4 in yeast and ATG4 A–D in mammals), resulting in the exposure of a glycine residue (Kirisako *et al.*, 2000). This C-terminal glycine is then adenylated by ubiquitin-activating enzyme (E1)-like ATG7 in the presence of adenosine triphosphate (ATP). ATG8 is subsequently transferred to the active site cysteine residue of ATG7, followed by the transfer to the catalytic cysteine residue of ubiquitin-conjugating enzyme (E2)-like ATG3. Finally, ATG8 is transferred from ATG3 to PE. The ATG12–ATG5–ATG16L1 complex exerts a ubiquitin ligase (E3)-like activity for ATG8-PE conjugation. Consequently, the C-terminal carboxyl group of ATG8 forms an amide bond with the amino group of the head group of PE. In the other ubiquitin-like system, the ubiquitin-like ATG12 covalently interacts with ATG5 through sequential reactions by ATG7 and E2-like ATG10 (Mizushima *et al.*, 1998). ATG12–ATG5 conjugation is irreversible, whereas ATG8s can be deconjugated from PE by ATG4 (Kirisako *et al.*, 2000; Tanida *et al.*, 2004).

Membrane-bound ATG8 regulates multiple steps in the autophagic process by recruiting effector proteins carrying the ATG8-interacting motif (AIM in yeast and LC3-interacting region [LIR] in mammals) to autophagosomes (Mizushima, 2020). For example, many autophagy adaptors such as p62 (also known as sequestosome-1; SQSTM1) have at least one LIR motif and one ubiquitin-binding domain, and, therefore, these adaptors link ubiquitinated cargos and the autophagic membrane (Fig. 6A) (Kirkin and Rogov, 2019; Lamark and Johansen, 2021; Vargas *et al.*, 2023). Tethering factors such as PLEKHM1 and EPG5 also possess a LIR motif and mediate the tethering between autophagosomes and lysosomes (McEwan *et al.*, 2015; Wang *et al.*, 2016). The binding of ATG8 to membranes also causes the deformation of membranes (Maruyama *et al.*, 2021), and this activity may promote autophagosome formation.

Mammalian ATG8 is also conjugated to phosphatidylserine (PS) during the conjugation of ATG8 to endosomal single membranes (CASM), which is a process induced by lysosomotropic reagents, LC3-associated phagocytosis, or influenza A virus infection (Fig. 1J) (Durgan *et al.*, 2021). The functional difference between ATG8-PE and ATG8-PS is their sensitivity to ATG4 proteins. ATG8-PS is resistant to cleavage by ATG4B but is susceptible to deconjugation by ATG4D *in vitro* when compared to ATG8-PE.

It had been widely thought that the conjugation of ATG8 to phospholipids is exceptional among the ubiquitin family proteins and that all other ubiquitins are conjugated to proteins. However, this is no longer considered to be accurate. Ubiquitin has also been observed to be conjugated to PE in the endosomal and vacuolar (or lysosomal in human) membranes (Sakamaki *et al.*, 2022). PE ubiquitination is detected in budding yeast, human cells, and baculoviruses. Similar to the ATG8 conjugation system, the C-terminal glycine residue of ubiquitin covalently binds to PE. PE ubiquitination is catalyzed by the conventional ubiquitination enzymes, including Uba1 (E1), Ubc4 and Ubc5 (E2s), and Tul1 (E3) in yeast (Fig. 6B) (Sakamaki *et al.*, 2022). The endosomal and vacuolar distribution of ubiquitinated PE is likely determined by the localization of Tul1, which is present mainly in endosomes and the vacuole. It is currently unknown how Tul1 recognizes PE as a substrate. PE ubiquitination is also reversible, and ubiquitin is removed from PE by the deubiquitinating enzyme Doa4. Ubiquitinated PE can recruit the membrane scission complex ESCRT *in vitro* and thus may be involved in intraluminal vesicle formation in cells. Ubiquitinated PE accumulates upon blocking vacuolar degradation, which suggests that ubiquitinated PE is concomitantly incorporated into intraluminal vesicles and delivered to the vacuole. In addition, other ubiquitin-like proteins NEDD8 and ISG15 are also conjugated to phospholipids, although their phospholipid substrates, localizations, and functions are unknown (Sakamaki *et al.*, 2022). Thus, covalent binding to phospholipids is likely a general characteristic of ubiquitin-family proteins.

Another example of ubiquitin-lipid conjugation is also observed during the autophagic removal of invading pathogens. Lipopolysaccharides on the *Salmonella* surface are ubiquitinated by the ubiquitin ligase RNF213 (also known as mysterin) in host cells (Otten *et al.*, 2021). Ubiquitin is likely conjugated to the lipid A moiety of LPS via an ester bond. Ubiquitination of LPS serves as a signal that recruits the autophagic machinery for clearance of the invading *Salmonella*. These findings suggest that ubiquitin can be conjugated to different types of lipids as well as proteins.

## Concluding remarks

Previous studies have uncovered the mechanisms and cellular and physiological roles of classical protein lipidation, including *N*-myristoylation, *S*-palmitoylation, *S*-prenylation, and GPI-anchoring. Recent studies have also uncovered previously unrecognized protein–lipid conjugation, including lysine *N*-fatty acylation, aspartic acid cholesterylation, and ubiquitin conjugation to phospholipids and LPS. More types of protein–lipid conjugation may be discovered in the future.

As described in this review, proteins can be linked to various types of lipids. Chemical biology and mass spectrometry as well as biochemical approaches have greatly contributed to the identification of protein–lipid conjugation. Click chemistry-based approaches using lipid analogs have become a powerful method to detect lipid-modified proteins (Gao and Hannoush, 2018; Kong *et al.*, 2019). It is estimated that more than 1,000 different molecular species of lipids exist in cells (van Meer *et al.*, 2008). There may be more types of protein–lipid conjugation yet to be identified. This field may extend to research in prokaryotes. However, the methods to identify lipids that are conjugated to proteins are not well established. Therefore, it is important to develop new tools and techniques to study lipids that covalently bind to proteins. Lipidomics may be applied to detect such species (Wang *et al.*, 2019).

Protein–lipid modification studies have focused mainly on the regulation of membrane localization of proteins, but several pieces of emerging evidence suggest the conjugation of proteins to lipids also regulates the dynamics of organellar membranes. Molecular dynamics simulation and theoretical modeling, in combination with *in vitro* biochemical experiments using artificial membranes or vesicles, have the potential to advance our understanding of the effect of protein–lipid conjugation on membrane morphology and dynamics.

Because protein–lipid conjugation is involved in essential cellular processes, including signaling and trafficking, the dysregulation of these modifications could be associated with various diseases, including cancer and infection. Therefore, protein lipidation has been considered a therapeutic target for these diseases (Chen *et al.*, 2018). Various inhibitors targeting lipid conjugation and deconjugation have been developed. As described in this review, fatty acyl conjugation on lysine residues was recently discovered, but the enzymes responsible for these modifications are poorly understood. Because the substrates are important for signaling pathways that are associated with cell proliferation, it is important to elucidate the catalytic mechanisms of these previously underrecognized modifications for the development of therapeutic agents, for example, against cancers.

## Declaration of Interests

The authors declare no competing interests.
